# Mosquito Vector Production across Socio-Economic Divides in Baton Rouge, Louisiana

**DOI:** 10.3390/ijerph18041420

**Published:** 2021-02-03

**Authors:** Rebeca de Jesús Crespo, Madison Harrison, Rachel Rogers, Randy Vaeth

**Affiliations:** 1 Department of Environmental Sciences, Louisiana State University, Baton Rouge, LA 70803, USA; rroge42@lsu.edu; 2 Health Sciences Center, School of Public Health, Louisiana State University, New Orleans, LA 70112, USA; mhar29@lsuhsc.edu; 3 East Baton Rouge Parish Mosquito Abatement and Rodent Control, Baton Rouge, LA 70807, USA; rvaeth@brla.gov

**Keywords:** mosquito vectors, *Aedes albopictus*, *Culex quinquefasicatus*, adjudicated properties, pest abatement requests, discarded tires

## Abstract

We investigated the role of socio-economic factors in the proliferation of mosquito vectors in two adjacent but socio-economically contrasting neighborhoods in Baton Rouge, LA, USA. We surveyed mosquito larvae habitat, mosquito larvae, and adult mosquitoes during the summer of 2020. We also evaluated the number of requests for mosquito abatement services in the years preceding the study for each area. While we did not find differences in terms of the most abundant species, *Culex quinquefasicatus* (F_1,30_ = 0.329, *p* = 0.57), we did find a higher abundance of mosquito habitats, particularly discarded tires, as well as larvae (z = 13.83, *p* < 0.001) and adults (F_1,30_ = 4.207, *p* = 0.049) of the species *Aedes albopictus* in the low-income neighborhood. In contrast, mosquito abatement requests were significantly higher in the high socio-economic neighborhood (z = −8.561, *p* < 0.001). This study shows how factors such as adjudicated properties, discarded tires and pest abatement requests can influence the abundance of mosquito vectors, disproportionately affecting low-income groups. This study also highlights how *Aedes* spp. may be better indicators than *Culex* spp. of socio-economic differences between nearby neighborhoods, due to their short flight range and habitat preferences, and this should be considered in future studies attempting to detect such disparities in the future.

## 1. Introduction

Container-breeding mosquitoes such as *Aedes albopictus* and *Culex quinquefasciatus* are important vectors of diseases such as Zika and West Nile virus, respectively. The proliferation of these vectors in residential neighborhoods may be linked to a variety of environmental and socio-economic factors that influence habitat availability and suitability. Important environmental drivers include temperature, precipitation, and vegetation cover, while socio-economic drivers are often associated with residential decay [1,2]. One of the main factors driving residential decay is the proportion of adjudicated properties and subsequent home abandonment. Abandoned homes can harbor mosquito breeding sites when water enters through the damaged infrastructure, or if the home has an abandoned water source, such as Jacuzzis or swimming pools [3]. Abandoned properties may also become the focus of illegal waste disposal practices within a neighborhood, further increasing mosquito habitat availability. Aside from abandonment, other factors may also vary in relation to socio-economic gradients that can be directly tied to public health disparities. Services associated with education, quality housing, access to healthy food, and walkability tend to be lower in low-income neighborhoods according to previous studies [4].

While previous studies have addressed the role of socio-economic disparities and mosquito-borne disease risk, the results of these studies are inconclusive and often demonstrate non-linear relationships [1,5]. Therefore, the relationships between poverty and mosquito disease risk may be context dependent and vary in relation to a number of local factors such as climate variability. To our knowledge, only one study [6] has addressed this question in Louisiana (LA), a state that provides ideal climatic conditions for many of the known mosquito vector species, and that is also one of the poorest states in the United States. In New Orleans, LA, Moise et al. [6] found that socio-economic factors, including neighborhood abandonment indicators, were not associated with the abundance of the mosquito *Culex quinquefasciatus*. This study, however, only focused on adult specimens, and overlooked larvae habitat availability and larvae abundance. The study also excluded *Aedes* species from the analysis. This limitation is important because *Aedes* species are the main vectors of some of the most prevalent mosquito-borne infections known to date [7,8], including Zika, dengue, chikungunya, and yellow fever. Understanding their dynamics is an important aspect of assessing mosquito risk [9].

In this study, we seek to evaluate if socio-economic differences between two adjacent neighborhoods lead to differences in mosquito abundance. Our study focused on two adjacent but socio-economically contrasting neighborhoods in Baton Rouge, LA, USA. We hypothesized that the lower socio-economic neighborhood will have a greater abundance of mosquito vectors, mainly due a higher proportion of abandoned properties, which may be the focus of illegal waste disposal. We test this hypothesis by conducting mosquito larvae and mosquito adult surveys in the two neighborhoods during the summer of 2020. The results of our study help determine the role of abandonment, illegal waste disposal, and pest control services on mosquito vector proliferation, and whether these factors lead to disproportionate health risks in low socio-economic neighborhoods.

## 2. Materials and Methods

### 2.1. Study Site

This study was located in the East Baton Rouge Parish, Baton Rouge, Louisiana. Baton Rouge is the capital of Louisiana and is located to the south eastern side of the state along the banks of the Mississippi river. Elevation ranges from 56 to 62 feet above sea level [10]. Population density is estimated at 2982.5 per square mile, total area covers 76.96 square miles, and median household income is estimated to be $41,761 per year [11,12]. Climate is humid subtropical, with rainfall averaging 55.5 inches per year, and temperature averages ranging from 51.7 to 83 °F [13].

**Figure 1:**
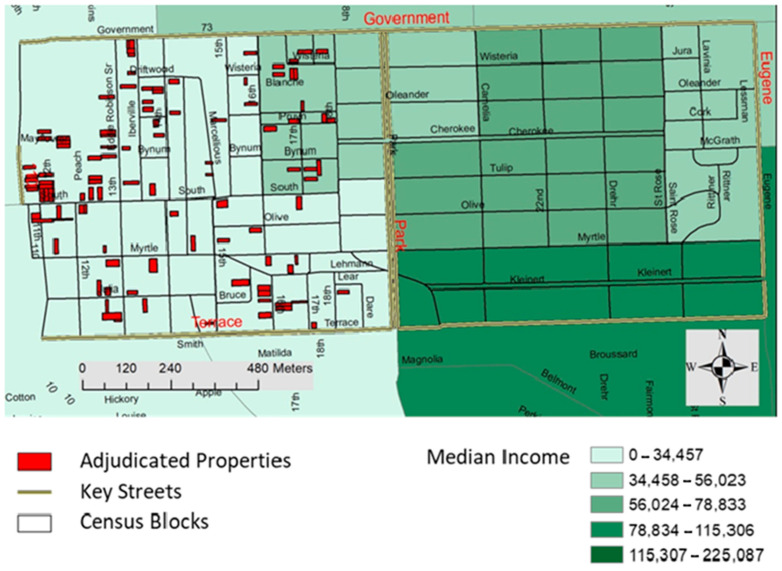
Study area in the Garden District neighborhood, Baton Rouge, LA, showing census block groups by median income, overlaying census blocks, key streets and adjudicated properties. Sources: adjudicated properties from Open Data Baton Rouge (2021); median income from the Amrican Community Survey (2017).

The selected neighborhood area within East Baton Rouge Parish is delimited by Government Street to the north, Terrace Street to the south, Eugene Street to the east and the I-10 ramp to the west (Figure 1). This neighborhood is divided in two distinct zones, to the east and to the west of Park Boulevard. Median income is higher on the east side ($66,319/year), than on the west side ($31,879/year), which is why these two neighborhood sectors are referred to as high-income and low-income neighborhoods hereinafter. The median income of the low- and high-income neighborhoods is also below and above (respectively) that of the city of Baton Rouge [11,12], and therefore these classifications are more broadly representative of socio-economic divides in the city. Population density, age and total area are similar between the high- and low-income neighborhoods, although the number of blocks is slightly higher in the low-income area (Table 1).

The high-income area is composed primarily of homes within the historic Garden District Neighborhood. The Garden District Neighborhood includes Roseland Terrace, which was the first subdivision in Baton Rouge, as well as Kleinert Terrace and Drehr Place, all of which date back to the first half of the 1900s, with planning and architecture reflecting the ideals of the “Garden City Movement” [14]. The low-income area is composed primarily of the Old South Neighborhood, a historically African American neighborhood, with landmarks such as the Lincoln Theater, which served as a cultural and entertainment center for African Americans during the civil rights era [15,16]. The Old South Neighborhood, while historically important, has experienced a steady decline in residents and an increase in urban blight in the last few decades [16]. For this reason, currently, the neighborhood is the focus of revitalization plans by multiple stakeholder groups [17].

### 2.2. Pest Abatement Requests

We obtained a list from the East Baton Rouge Parish Mosquito Abatement and Rodent Control (EBRP-MARC) with contact information and the number of calls that they received requesting mosquito spraying services from 2017 to 2019. These requests represent data on completed inspections that resulted in an application of pesticide in the requesting household. Aside from responding to these calls, the EBRP-MARC also conducts monitoring of pest activity and coordinated pesticide application in areas perceived to be at high risk. However, responses to pest abatement requests represent an additional service provided by this program.

**Figure 2:**
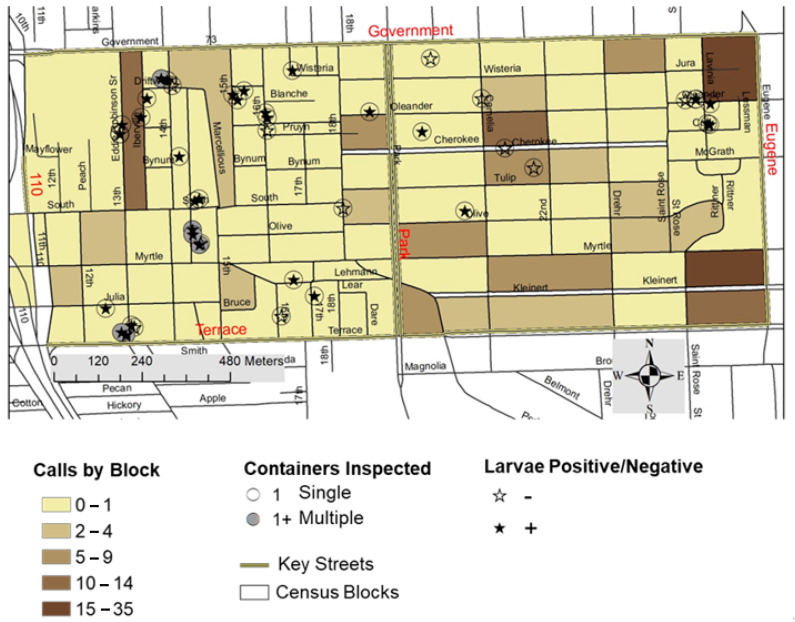
Service request calls and results from larvae survey showing locations with larvae positive and larvae negative containers, as well as locations of inspected containers, indicating places with more than one container (1+). All of the multiple (1+) containers were discarded tires.

Pest abatement request data was used to identify possible participants for adult mosquito trapping (see below), and to compare the number of pest abatement requests between the neighborhoods (Figure 2). Calls were summarized at the census block level, eliminating any personal identifiable information from the study.

### 2.3. Mosquito Larvae Survey

We surveyed all census blocks in the study area on three occasions during the summer of 2020 (2 June 2020, 14 July 2020, and 4 September 2020). Two people conducted the first two surveys, and one person conducted the last survey. Rainfall for the sampling period, including the month prior to the sampling events, was approximately 27.16 inches, with the month of July being the wettest (8.91 inches) and the month of September being the driest (1.81 inches) [13]. Temperature during the sampling period ranged from a maximum of 93.5 °F to a minimum of 64 °F [13].

We standardized our sampling effort by limiting our search of mosquito larvae habitats to two hours in each of the two neighborhoods. We inspected all potential larvae habitats (discarded tires, plant pots, water baths, etc.) in publicly accessible locations. We determined whether the inspected containers had water and contained larvae to calculate a neighborhood level container index (% of water holding containers with larvae) [9]. For all water-filled containers, we estimated total water volume and temperature. For larvae positive containers, we sampled approximately half a liter of water containing larvae for further identification of larvae species using morphological keys. The GPS location of inspected containers (positive or negative for larvae) was recorded for later spatial analysis (Figure 2).

### 2.4. Adult Mosquito Survey

Adult mosquito trapping was performed at 11 locations (5 in the low-income neighborhood and 6 in the high-income neighborhood) on three occasions during the summer of 2020 (30 June 2020, 31 July 2020, and 4 September 2020). We used BG Sentinel traps, baited with a BG Lure (BioGents, Regensburg, Germany), for a period of 24 h on each of the sampling occasions.

The location of the traps included an abandoned home in the low-income neighborhood and an empty lot with trash accumulation in the high-income neighborhood. The rest of the traps were located at private residences in an outdoor and covered location. Volunteer households were identified by recommendation from community organizations, knocking on people’s doors, and by identifying individuals that had requested the pest control services of the EBRP-MARC. For the latter source of volunteers, we made sure to include an equal number of houses in each neighborhood that had requested these services (*n* = 2 on each side) to avoid skewing results.

### 2.5. Statistical Analysis

For our larvae survey, we combined data from the three sampling events and used census blocks as our analytical unit. We used neighborhood (high-income (0) and low-income (1)), as a binary explanatory variable and analyzed differences in total larvae and total larvae by species using a Poisson linear regression model. We also evaluated differences in the number of calls to request pest abatement services between 2017–2019 between the high-income and low-income neighborhoods (0,1, respectively) using a Poisson linear regression.

For our adult survey, we combined the samples collected on the three occasions and used analysis of variance, using neighborhood (0,1) for the main effect, and total adults by species as the response. For the ANOVA, we log transformed our data to meet normality assumptions prior to analysis.

All analyses were conducted using R statistical software [18].

## 3. Results

### 3.1. Mosquito Abatement Data

There were a total of 177 pest abatement requests between 2017 and 2019 for the study area. Of these, the majority (*n* = 143, 80.8%) were from the high-income neighborhood (Figure 2), and this difference in service requests was found to be statistically significant (z = −8.561, *p* < 0.001). While the official call data was not available at the time of this study, and therefore not used in the analysis, during the study period (June–September 2020), the EBRP-MARC conducted coordinated pesticide applications on two occasions in each neighborhood: on 10 June 2020 and 14 July 2020 in the low-income neighborhood, and on 15 July 2020 and 19 August 2020 in the high-income neighborhood. These abatement events would not affect larvae, and since they occurred at least two weeks apart from our adult sampling events (30 June 2020, 31 July 2020, and 4 September 2020), they should have not interfered with our adult sampling data. Also, these two abatement efforts are separate from those that would result from resident requests.

### 3.2. Mosquito Larvae Survey Results

We collected a total of 1619 larvae. This included 1030 *Cx. quinquefasciatus* and 546 *Ae. albopictus*, which were the two dominant species found. The high-income area had 38.64% of the *Cx. quinquefasciatus* collected, and 10.98% of the collected *Ae. albopictus*. The low-income area had 61.36% of the *Cx. quinquefasciatus* larvae and 89.01% of the *Ae. albopictus* larvae.

Aside from these two dominant species, we also found *Toxorhynchites rutilus* (11 individuals, low-income area), *Culex coronator* (29, low-income area), *Culex territans* (2, high-income area), and *Psorophora* sp. (1, low-income area). The presence of *Tx. rutilus*, a predatory species to other mosquito larvae, suggests that habitat conditions are adequate for its potential use as a biological control agent in this study region.

A total of 44 container habitats were found and surveyed, 34 of which (77.27%) were discarded tires. Discarded tires were more abundant in the low-income area, accounting for 71.05% of the number of discarded tires found. Out of these, seven containers (all discarded tires) were dry at the time of inspection during the September sampling event, which reflects the low rainfall conditions during this and the prior month of August. Two of these dry tires were located in the high-income neighborhood, and the rest were located in the low-income neighborhood. The remaining 37 containers had water at the time of inspection and were sampled for larvae and habitat traits. Table 2 describes these containers in terms of the presence/absence of larvae, container type, water temperature, and water volume.

In the low-income neighborhood, we found several piles of discarded tires, in which case we only selected one tire to survey from each pile. Selection was based on accessibility and water volume. Therefore, the number of tires presented here is an underestimation of the total number of tires in the low-income neighborhood. Figure 2 highlights areas where multiple tires were found.

Our statistical analysis showed that the low-income neighborhood had significantly higher numbers of *Ae. albopictus* larvae (z = 13.83, *p* < 0.001), and higher numbers of total larvae (z = 9.83, *p* < 0.001) than the high-income neighborhood. Accordingly, the container index value for the low-income neighborhood was higher (CI = 0.96) than for the high-income neighborhood (CI = 0.70). We found no significant difference in terms of total *Cx. quinquefasciatus* larvae between the two neighborhoods (z = −0.20, *p* = 0.841).

### 3.3. Adult Survey Results

Two of the homes in the high-income neighborhood dropped out of the study in the second and/or third sampling events. These homes were substituted by a neighboring home (150 m or less distance from the original home). One home from the low-income neighborhood was not available for sampling during the second sampling event. This home was not substituted as we were not able to find another home to participate that was within 150 m of the original home. In total, we collected 607 adult mosquitoes; 44.32% of these were *Cx. quinqefasciatus*, 40.03% were *Ae. albopictus*, and 15.65% were *Anopheles* (spp). Details on the abundance and proportion of these species per neighborhood are provided in Table 3.

ANOVA results suggest that there was no significant difference between neighborhoods in terms of total *Cx. quinquefasciatus* adults (F_1,30_ = 0.329, *p* = 0.57) or total number of adult mosquitoes (F_1,30 =_ 1.371, *p* = 0.251) in the study area. We found a significantly higher number of *Ae. albopictus* adults in the low-income neighborhood than the high-income neighborhood (F_1,30_ = 4.207, *p* = 0.049).

## 4. Discussion

This study is the first to evaluate the role of socio-economic drivers on the presence of larvae and adult mosquito vectors of *Culex* and *Aedes* species in the state of Louisiana, a state with ideal climatic conditions for mosquito vectors, and which has some of the highest poverty rates in the country. While we found no difference in terms of larvae and adults of the species *Cx. quinquefasciatus*, we did find more *Ae. albopictus* (larvae and adults) in the low-income neighborhood. This difference was likely associated with the higher presence of container habitats in the form of discarded tires, and other signs of urban disrepair such as a greater number of adjudicated properties in the low-income neighborhood.

Our study results are similar to another recent study in New Orleans, LA, which focused only on *Cx. quinquefasciatus* adults and found no association between this species and socio-economic or neighborhood abandonment indicators [6]. There are several reasons why *Cx. quinquefasciatus* may not be directly affected by socio-economic factors. Unlike *Aedes* species, *Cx. quinquefasciatus* commonly uses a variety of containers with large volumes of water, such as storm water treatment devices and catch basins [19], allowing the species to exist in locations where there are no waste management problems but where there are “functional” container habitats available. These functional containers may not differ much between high- and low-income neighborhoods. Second, the flight range of *Cx. quinquefasciatus* can span up to 2 km [20]. This allows for easier crossing between socio-economic divides relative to other shorter dispersing species, such as *Aedes* species. Lastly, *Cx. quinquefasciatus* adults have been found to be less susceptible to certain types of frequently used pesticides (e.g., permethrin) than *Aedes* or *Anopheles* mosquitoes [21], suggesting that greater pesticide application may not necessarily result in long term control of this mosquito locally. Accordingly, even though pest abatement control service calls were more frequently made from the high-income neighborhood than the low-income neighborhood, *Cx. quinquefasciatus* were found in similar abundances in both locations.

There was a higher number of pest abatement requests from the high-income neighborhood even though the total number of *Ae. albopictus* adult and larvae, the total number of larvae, and the number of larvae positive containers were all higher in the low-income neighborhood. Similar results have been found in previous studies. Feigenbaum et al. [22] found that in Boston, Massachusetts, high-income census tracts made and received more service requests to government than their low-income counterparts, and this was not related to a higher need for services in the high-income group. Their findings could be explained by the use of cell phone apps to place the requests using smartphones, which may be less frequently owned by low-income individuals. While the EBRP-MARC does not operate based on smartphone apps, finding the information to place a request call is facilitated by an internet connection to access the agency’s website, which may represent a barrier to low-income households. Another reason for service request disparities may include a lack of trust in government services, and a lack of information about the options available for residents [22]. In addition, exposure to higher levels of “neighborhood physical disorder”, such as abandoned homes, empty lots, and trash, is considered to be a pathway towards low individual self-esteem and civic engagement [23], which may in turn translate to less motivation and empowerment for requesting government services. Lastly, low-income areas exposed to multiple stressor environments [24] may be taxed by concerns about other social or environmental stressors, and this may serve as a barrier to focusing attention on mosquito problems, which may be perceived as less of a threat. Interestingly, service requests to EBRP-MARC tend to be comparatively higher in the low-income neighborhood vs. the high-income neighborhood for rodent control (EBRP-MARC, personal communication). While the total number of calls for rodents are still lower than for mosquitoes on both sides, and the disparity in requests for other pest control services is beyond the scope of the present study, this example helps illustrate competing priorities of residents in the low-income neighborhood that may be further exacerbated by the overall level of disrepair of their surrounding environment. In sum, given the greater mosquito production risk found in the low-income area, our study suggests that social factors, such as greater exposure to neighborhood stressors, mosquito-related perceptions or knowledge-related barriers, lead to disparate claims for mosquito services, and this requires greater investigation.

We found a higher number of larvae positive containers and a higher abundance of *Ae. albopictus* in the low-income neighborhood. Unlike *Cx. quinquefasciatus*, *Ae. albopictus* has a shorter flight range (150–200 m), is more often found in artificial containers with high evaporation rates such as styrofoam cups and discarded snack bags [25], and is more successful at exploiting discarded tires as a habitat through the mosquito production season [18]. These qualities likely make this and other *Aedes* species, such as *Ae. aegypti*, better indicators of socio-economic drivers of mosquito vector habitat, as these species are more strongly associated to above-ground artificial containers with high evaporation rates [25], a characteristic of urban waste. However, this study only surveyed mosquito larvae habitats in easily accessible public spaces. This approach does not fully characterize differences in habitat availability within individual properties, or provided by underground culverts, catch basins and other storm water management systems. While our adult mosquito sampling partly captures production from these “hidden” sources of mosquito breeding sites, we caution that the overall number of larvae habitats may be underestimated and that the differences between neighborhoods stemming from these other sources should be studied further.

Of the diseases that are carried by *Aedes* species, dengue has been locally transmitted in Louisiana. Although the last time this occurred was in the 1940s [26], there has been local transmission of dengue reported more recently in states like Texas [27] and Florida [28], demonstrating that there is a potential for re-emergence of this disease, and introduction of other diseases like Zika and chikungunya in other parts of the US [29]. Therefore, understanding the factors that contribute to the proliferation of these vectors is of great importance.

Our study has several limitations. First, it compared only two neighborhoods within the city of Baton Rouge, and therefore the results are not necessarily representative of trends that occur more broadly in the city, or other parts of East Baton Rouge Parish. Future studies should address this question more widely to identify if there are specific locations across the city where disparities are more apparent, or where disparities do not occur. Nevertheless, the spatial scale that influences mosquito habitat suitability tends to be small (30–100 m) [30,31], and addressing this question on a per neighborhood basis as we have done in this study is also adequate. Second, we conducted this survey during one summer period on three occasions, and therefore it does not fully portray temporal variations in mosquito abundance. However, by conducting this study during summer/early fall, we attempted to capture the period where mosquito activity is the highest [8,32]. As such, our data is representative of differences in mosquito abundance between these two neighborhoods. Lastly, our study only focused on one method of capturing adult mosquitoes (BG Sentinel traps, and BG Lures). Supplementing this method with other approaches such as CO_2_ baits, gravid traps, and light traps would have allowed us to capture a wider variety of mosquitoes, which would provide more information about differences between neighborhoods.

Our results likely underestimate the actual abundance of mosquito and mosquito habitat availability in the low-income neighborhood. As mentioned earlier, we did not sample the entirety of the larvae habitats found during our survey, and we had fewer households participating in the adult mosquito collection effort in the low-income neighborhood. During our neighborhood survey, the abundance of discarded tires (Figure 2), and abandoned properties (Figure 1) was associated with a higher abundance of mosquito habitats (Figure 2). Adjudicated properties were focal locations for illicit trash disposal practices, and discarded tires were the most abundant container type found.

In Baton Rouge, several efforts have tried to address urban blight problems such as the ones described here. The Mow to Own program provides incentives for purchasing an adjudicated property to adjacent neighbors that have contributed to its maintenance for a period of one year [33]. Likewise, in 2018, the EBRP-MARC received a $605,000 grant from the Center for Disease Control and Prevention to build a tire shredder for reducing mosquito habitats across the city of Baton Rouge [34]. While the success of these recent efforts is yet to be determined, our study highlights the importance of such initiatives to address environmental justice problems in Baton Rouge, and more broadly Louisiana. From our study, it is evident that issues of blight are leading to disproportionate mosquito vector disease risk in low socio-economic neighborhoods and that blight reduction efforts should continue for the benefit of public health.

## Figures and Tables

**Table 1:** General characteristics of the neighborhoods sampled. Source: American Community Survey (2017), US Census (2010), and Open Data Baton Rouge (2021).

Characteristics	Low-Income	High-Income
Number of blocks	56	44
Total area	669,064 m^2^	667,087 m^2^
Average block area	11,947 m^2^	15,161 m^2^
Average population per block	27.14	37.20
Total population	1520	1656
Population density	0.002 pp/m^2^	0.002 pp/m^2^
Average median income	$31,879	$66,319
Average median age	33.5	34.2

**Table 2:** Description of all water-filled containers found in our study area.

Neighborhood	Container Type (−, +) *	Number of Containers	Average Temperature (°F)	Average Volume (L)	Container Index
High Income	Total	10	85.21	1.52	0.7
	Bird bath (−)	1	89.42	2.37	
	Bucket (−)	2	91.22	2.25	
	Bucket (+)	1	83.48	0.24	
	Tire (+)	5	83.3	1.29	
	Toy (+)	1	80.24	1.66	
Low-Income	Total	27	79.81	2.05	0.96
	Tire (−)	1	79.88	0.24	
	Tires (+)	21	78.44	2.36	
	Bucket (+)	2	83.21	2.37	
	Food Container (+)	1	83.66	0.47	
	Cup (+)	1	93.38	0.35	
	Trash (other) (+)	1	83.84	0.12	

* (−, +) denotes larvae absent or larvae present, respectively.

**Table 3:** Adult mosquito survey results by neighborhood (HI: high-income; LI: low-income).

Adult Mosquito Taxa	Total			% of Total	Average per Trap	
	HI	LI	Grand Total		HI	LI
*Culex quinquefasciatus*	169	100	269	44.32	9.39	7.14
Female	49	19	68	11.20	2.72	1.36
Male	120	81	201	33.11	6.67	5.79
*Aedes albopictus*	71	172	243	40.03	3.94	12.29
Female	41	93	134	22.08	2.28	6.64
Male	30	79	109	17.96	1.67	5.64
*Anopheles spp.*	48	47	95	15.65	2.67	3.36
Female	0	0	0	0.00	0.00	0.00
Male	48	47	95	15.65	2.67	3.36
Total	288	319	607	100.00		

## Data Availability

Data generated in this study is available upon request.
